# Effectiveness and feasibility of a mindful leadership course for medical specialists: a pilot study

**DOI:** 10.1186/s12909-020-1948-5

**Published:** 2020-02-04

**Authors:** Wendy M. Kersemaekers, Kiki Vreeling, Hanne Verweij, Miep van der Drift, Linda Cillessen, Dirk van Dierendonck, Anne E. M. Speckens

**Affiliations:** 1 Radboudumc Center for Mindfulness, Department of Psychiatry, Radboudumc, P.O. Box 9101, 6500 HB Nijmegen, The Netherlands; 2 Department of Respiratory Medicine, Radboudumc, Nijmegen, The Netherlands; 3 Donders Institute for Brain, Cognition and Behaviour, Radboud University, Nijmegen, The Netherlands; 4 Department of Organisation and Personnel Management, Rotterdam School of Management, Erasmus University, Rotterdam, The Netherlands

**Keywords:** Mindfulness, Burnout, Well-being, Leadership, Continuing medical education, Feasibility, Medical specialists

## Abstract

**Background:**

Medical specialists experience high levels of stress. This has an impact on their well-being, but also on quality of their leadership. In the current mixed method study, the feasibility and effectiveness of a course Mindful Leadership on burnout, well-being and leadership skills of medical specialists were evaluated.

**Methods:**

This is a non-randomized controlled pre-post evaluation using self-report questionnaires administered at 3 months before (control period), start and end of the training (intervention period). Burn-out symptoms, well-being and leadership skills were assessed with self-report questionnaires. Semi-structured interviews were used to qualitatively evaluate barriers and facilitators for completion of the course.

**Results:**

From September 2014 to June 2016, 52 medical specialists participated in the study. Of these, 48 (92%) completed the course. Compared to the control period, the intervention period resulted in greater reductions of depersonalization (mean difference = − 1.2, *p* = 0.06), worry (mean difference = − 4.3, *p* = 0.04) and negative work-home interference (mean difference = − 0.2, *p* = 0.03), and greater improvements of mindfulness (mean difference = 0.5, *p* = 0.04), life satisfaction (mean difference = 0.4, *p* = 0.01) and self-reported ethical leadership (mean difference = 0.1, *p* = 0.02). Effect sizes were generally small to medium (0.3 to 0.6) and large for life satisfaction (0.8). Appreciation of course elements was a major facilitator and the difficulty of finding time a major barrier for participating.

**Conclusions:**

A ‘Mindful Leadership’ course was feasible and not only effective in reducing burnout symptoms and improving well-being, but also appeared to have potential for improving leadership skills. Mindful leadership courses could be a valuable part of ongoing professional development programs for medical specialists.

## Background

High work demands and minimal ability to control put high pressure on medical specialists, who often have to combine a leadership role with being a clinician. This can lead to stress and burnout symptoms [1, 2], which not only impair their own well-being, but are also related to decreased patient safety [3, 4] and reduced quality of care [5, 6]. High stress levels do not only affect the well-being of the medical specialist and his or her patients, but also affect the entire team of the medical specialist; high stress is related to decreases in ability to collaborate and the quality of their leadership [5, 6]. Low quality leadership is associated with more burnout and less satisfaction among physicians in the whole department [7, 8]. Therefore, it is essential that medical specialists have skills to deal with stress and high pressure, and to provide good quality leadership.

The importance of development of these non-clinical competencies in addition to clinical competencies is increasingly recognized [9]. The CanMEDS and professional organizations such as the Accreditation Council on Graduate Medical Education defined competencies such as self-regulation, reflective practice, resilience, leadership, and clear communication as key competencies of the professional role of physicians [9]. These non-clinical competencies are now recognized as fundamental to medical practice and healthcare, and are starting to be integrated into the formal medical curricula. However, besides integration of non-clinical competencies in the education of medical students and residents, training in these competencies should also be offered as part of the ongoing education throughout the medical professional’s career [10].

A course ‘Mindful Leadership for Medical Specialists’ might support medical specialists in developing these competencies. Mindfulness is defined as intentionally paying attention and being aware of moment by moment experiences in a non-judgmental and friendly way [11]. Mindfulness may improve different aspects of self-regulation, i.e. attention regulation, emotion regulation and self-awareness, which are all relevant building blocks for coping with stress and effective leadership [12, 13].

Several publications described the potential relevance of mindfulness for medicine regarding quality of care [14], physician resilience [5] and for leadership [13, 14]. Mindfulness has been shown to be effective in reducing stress and burnout symptoms, and improving quality of life in healthy individuals [15], including mixed groups of healthcare professionals [16], and physicians [17–20]. Qualitative studies also reported improved communication skills [21, 22], with patients reporting being better understood by their clinicians [21]. However, relatively few studies of health care professionals were conducted in medical specialists and/or leaders [16]. Moreover, little is known about the effects of mindfulness interventions on physicians’ leadership skills [23] and leadership in general [12]. Furthermore, data on the feasibility of completing a mindful leadership course for this profession is largely lacking [16]. Therefore, the current pilot mixed method study evaluated the feasibility and effectiveness of a mindful leadership course in medical specialists on burnout, well-being and leadership.

## Methods

### Design

The study consisted of a quantitative and qualitative part. The quantitative study evaluated the effectiveness of the ‘Mindful Leadership for Medical Specialists’ course using a non-randomized controlled pre-post evaluation in medical specialists using self-report questionnaires administered 3 months before the course (control period), and at the start and end of the course (intervention period). Qualitative semi-structured interviews were used to explore the facilitators and barriers for participating in the course.

According to the Dutch law (law Medical Scientific Research with humans, https://wetten.overheid.nl/BWBR0009408/2019-04-02), because of the non-invasive and non-experimental nature of the study and the minimal risks and burdens for participants, this study did not require review by The Medical Ethical Committee Arnhem/Nijmegen, the Netherlands. However, the principles of the Declaration of Helsinki were followed whenever applicable. Participants agreed to participate by signing an informed consent form, explicitly agreed to recording of the interviews and data were treated confidentially by anonymizing them before analyses.

### Procedure

Medical specialists were recruited from different hospitals in the eastern part of The Netherlands by mailings and via intranet of the hospital (purposive sampling). The first two cohorts participated in the Mindful Leadership course for free, whereas the last two cohorts also received (partial) financial compensation through use of their personal development budget.

Participants were invited to fill out questionnaires via an online survey system 1 week before the start of the course and after the last session of the course (intervention period). In addition, participants were invited for the control period from the second group onwards and if they had subscribed more than 2 months before start of the training (control period). Therefore, not all participants could contribute to the control period. Furthermore, some participants completed the control period, but did not take part in the intervention period.

A subset of the participants were invited for semi-structured interviews approximately 1 year after the course. Initially, participants were invited at random from the entire sample, subsequently more purposively by sampling medical leaders, participants who discontinued, and balancing male and female participants.

### Intervention

The Mindful Leadership for Medical Specialists course consisted of ten two-weekly group sessions of five hours. Sessions were scheduled from 4 to 9 pm with a break for dinner in between. Groups included 11 to 19 participants. The courses were taught by a senior medical specialist/mindfulness trainer together with a senior leadership trainer. The mindfulness trainers fulfilled the teacher criteria of the UK network for Mindfulness-Based Teacher Training Organizations [24].

The course contained the full 8-weeks Mindfulness Based Stress Reduction (MBSR) training (divided over sessions 1–10) as developed by Kabat-Zinn [11], including a variety of formal and informal meditation practices such as the body scan, sitting meditation, walking meditation, mindfulness yoga, 3 min breathing space and a silent day. In these practices, participants were instructed to focus their attention, for instance on the breath or the body, and to do this with a kind attitude towards themselves. In addition, psycho-education and experiential teaching sessions were offered on recognizing and managing stress. Participants were encouraged to practice mindfulness at home, 30–45 min per day. Home practice included formal meditation practice and the application of mindfulness in everyday life, for example by paying mindful attention to everyday activities. From session 6 onwards, ‘mindful communication’ was included, following the guidelines of ‘Insight dialogue’ by Gregory Kramer [25].

The course also explicitly addressed leadership. The leadership component consisted of explanations of general leadership theories in sessions 2, 3 and 6 (respectively Covey’s seven habits for leadership success [26], Hersey’s situational leadership [27], and Scharmer’s Theory U [28]). Explanations of these theories were placed in the context of mindfulness and participants were asked to reflect on how the new information could be applied, and to practice this in their daily work.

In sessions 4 and 5, experiential teaching sessions were offered on cognitive behavioral therapy. This included education on the cognitive model, i.e. how a situation can trigger automatic thoughts and concurring emotions, physical sensations and behavior, and how this relates to intermediate and core beliefs [29]. Participants were asked to develop their own cognitive model (session 4) and to define alternatives for their intermediate beliefs, preferably in relation to their role as leader (session 5). Homework consisted of the practical application of this in their work situation.

Sessions 8 and 9 included teachings and practices on compassion [30]. These included loving kindness meditation, the compassionate breathing space and/or bodyscan and experiencing and discussing how compassion relates to leadership.

### Quantitative study

#### Measures

Burnout was measured with multiple questionnaires to capture different aspects. We used the subscales emotional exhaustion, depersonalisation and personal accomplishment of the Utrecht Burnout Scale (UBOS-C) [31], which is a Dutch version of the Maslach Burnout Inventory to assess burn-out symptoms [32]. Furthermore, we assessed worry with the Penn State Worry Questionnaire Survey (PSWQ) [33]. Twelve items of the Work Home Interaction Nijmegen scale (SWING) were included to assess negative work-home and home-work interferences [34]. Psychological well-being was measured by using the Mental Health Continuum-short Form (MHC-SF) [35] and the Life Satisfaction Questionnaire (LSQ) [36]. In addition, the Self-Compassion Scale- Short Form (SCS-SF) was used to assess overall self-compassion and mindfulness (subscale) [37]. A self-report version of the Ethical Leadership at Work questionnaire (ELW) was used to assess ethical leadership with the subscales fairness, integrity, ethical guidance, people orientation, power sharing, role clarification, and concern for sustainability [38].

#### Analysis

Linear mixed model analysis (SPSS version 22 [39]) was used to estimate mean scores on the different time points and the mean changes within the pre-intervention (t0 and t1) and intervention period (t1 and t2).

Because two comparisons were tested (t0 and t1, and t1 and t2), Bonferroni correction for multiple testing was applied by multiplying *p*-values by two for the pre–post assessments within the intervention and pre-intervention period. The mean difference between the pre-intervention and intervention periods (intervention effect) was estimated by subtracting the effect in the pre-intervention period from the effect in the intervention period. Linear mixed models take into account the nesting of repeated measurements within individuals.

The model estimates mean outcome scores for each timepoint based on the available data, assuming that the participants consist of an a-select sample of a background population that we are interested in [40]. One advantage of this modelling is that all data points can be included in the analyses, independent of completeness of the data, thereby avoiding further selection of the data and subsequent selection bias.

Given the pilot character of the study, we presented *P* -values of < 0.1, < 0.05 and < 0.001 and considered these indicative for an effect deserving further investigation [41]. Furthermore, a Cohen’s *d* type of effect size was calculated based on the model estimated mean difference between the effect in the intervention and the control period divided by the square root of the variance between participants (intercept). In order to prevent selection bias, we included all participants in the analyses, independent of completion of the training or attendance.

As a quantitative indication for the feasibility of completing the course, completion rates were calculated based on the number of sessions attended.

### Qualitative study

Twenty-five medical specialists that participated in the Mindful Leadership course were contacted by email to ask them for participation in the qualitative study part. Seventeen medical specialists agreed to participated. All interviewees knew the rational of the current study due to the informed consent procedure, but they were not aware of the personal goals of the researchers.

The interviewers were WK and KV. WK is a senior researcher and mindfulness trainer, KV is a business consultant and mindfulness trainer. WK and KV expected beneficial effects of the mindfulness training on burn out and leadership. The interviewers did not have a relationship with the participants prior to their participation in the current study. Only the interviewer and the participants were present during the interview. The participants did not check the transcripts of the interviews, nor did they provide feedback on the findings.

The average interview time was up to an hour. Interviews took place at a location preferred by the respondent, which was mostly in the workplace. Interviews were recorded with a voice recorder, there were no field notes made. The questions related to feasibility were:

- How feasible was it for you to participate in this course?
- What were facilitators and barriers to complete the course?

Continued open questioning was used to deeply understand the provided answers. Perceived effects of the course on personal and professional functioning and leadership skills were also explored, and are reported in a separate publication [42]. However, if responses to these questions were considered to be facilitators or barriers to participating in the course, they were included in the qualitative analysis of the feasibility. Saturation was reached for perceived effects of the course (see 42). The interview questions were not pilot tested.

All interviews were transcribed and analyzed in Atlas Ti version 7 by using thematic analysis. Familiarization with the data and independent coding was done by two different researchers (WK and HvR, or WK and KV). The codes were discussed and agreed upon by the same two researchers. Next, the codes were checked for similarity, and similar codes were grouped. Themes were defined and explored, and corresponding quotes were selected.

## Results

### Quantitative study

From September 2014 to June 2016, 4 groups of medical specialists (*N* = 52) were trained. In addition, 9 medical specialists contributed to the control period only. Response rates to the questionnaires were high, with complete responses for 19 (83%) of those who were invited to all three time points, 26 (90%) of those who were invited to the intervention period only (t0 and t1) and 7 (78%) of those invited to the control period only (t-1 and t0) (see Table 1). This resulted in available data of 59 participants and 134 completed surveys. Demographics and number of attended sessions are displayed in Table 2.

**Table 1:** Number of participants that were invited and responded to the surveys at different time points

Time points in the study	Invited	Completed all N (%)	Completed part N (%)	Completed nothing N (%)	Total # participants in database
Control+ intervention (t-1, t0 and t1)	23	19 (83%)	4 (17%)		23
Intervention (t0 and t1 only)	29	26 (90%)	3 (10%)		29
Control only (t-1 and t0)	9	7 (78%)	0 (0%)	2 (22%)	7
Total	61	52 (85%)	8 (13%)		59

*t-1* start of control period

*t0* end of control/start of intervention period

*t1* end of intervention period

**Table 2:** Characteristics of the participants (*N* = 59^a^)

Characteristic	
Gender (n, %)	
Male	20 (34%)
Female	39 (66%)
Age (mean, sd)	47.9 (8.0)
Marital status (n, %)	
Single	2 (3%)
Relationship, not living together	3 (5%)
Relationship, living together	11 (19%)
Married	41 (70%)
Divorced	1 (3%)
Children (n, %)	
None	7 (12%)
One	3 (5%)
More than one	48 (83%)
Specialism (n, %)	
Internal	30 (52%)
Surgical	16 (28%)
Supportive	12 (21%)
Years in practice (mean, sd)	13.3 (8.5)
Number work hours per week (mean, sd)	50.5 (9.7)
Number of sessions attended (n, %)^b^	
1–4 sessions	4 (7.7%)
5–6 sessions	4 (7.7%)
7–10 sessions	44 (84.6%)

^a^ One participant did not provide complete demographics

^b^ Excluding control-only participants (*N* = 7)

Results of the linear mixed models are displayed in Table 3. Compared to the control period, the intervention period resulted in great reduction in burnout. This was reflected in greater reductions of depersonalization, worry and negative interference of work with home. Emotional exhaustion and personal accomplishment improved during the intervention period and not during the control period, but the between period difference was not statistically significant. No difference was observed for the negative interference of home with work.

**Table 3:** Mean scores (standard error, SE) and mean differences (SE) between time points and mean differences (SE) between intervention and control period and their effects sizes

	Mean (SE)^a^ T-1	Mean (SE)^a^ T0	Mean (SE)^a^ T1	Mean difference T-1 to T0 (SE)	Mean difference T0 to T1 (SE)	Difference between periods	Cohen’s *d*
Burnout (UBOS)							
Emotional exhaustion	15.3 (1.2)	14.9 (1.0)	12.4 (1.1)	−0.4 (1.0)	−2.5 (0.8) ***	− 2.1 (1.4)	− 0.3
Depersonalization	5.0 (0.4)	5.1 (0.4)	4.1 (0.4)	0.1 (0.4)	−1.0 (0.3) ***	−1.2 (0.6)*	−0.6
Personal accomplishment	26.4 (0.8)	27.0 (0.7)	28.4 (0.7)	0.6 (0.6)	1.4 (0.5) ***	0.8 (0.8)	0.2
Trait of worry (PSWQ)	37.1 (1.8)	37.9 (1.6)	34.4 (1.6)	0.8 (1.4)	−3.5 (1.2)***	−4.3 (2.1)**	−0.4
Interference work and home (SWING)							
Negative work home interference	2.3 (0.1)	2.3 (0.1)	2.1 (0.1)	0.0 (0.1)	−0.2 (0.0) ***	−0.2 (0.1)**	− 0.5
Negative home work interference	1.2 (0.0)	1.2 (0.0)	1.2 (0.0)	0.0 (0.0)	0.0 (0.0)	0.0 (0.1)	0.0
Self Compassion (SCS-SF)	4.4 (0.1)	4.5 (0.1)	4.9 (0.1)	0.1 (0.1)	0.4 (0.1) ***	0.2 (0.2)	0.3
Mindfulness	5.2 (0.2)	5.0 (0.1)	5.3 (0.1)	−0.2 (0.1)	0.3 (0.1)**	0.5 (0.2)**	0.6
Mental Health (MHC)	3.0 (0.1)	3.1 (0.1)	3.4 (0.1)	0.1 (0.1)	0.3 (0.1)***	0.2 (0.2)	0.4
Life Satisfaction (LSQ)	5.0 (0.1)	4.9 (0.1)	5.1 (0.1)	−0.1 (0.1)	0.3 (0.1)***	0.4 (0.1)**	0.8
Ethical leadership at work (ELW)	3.8 (0.1)	3.8 (0.0)	4.0 (0.0)	0.0 (0.0)	0.2 (0.0)***	0.1 (0.1)**	0.4

^a^ Estimated marginal means and standard errors

*p < 0.1, ** *p* < 0.05, *** *p* < 0.01

Psychological well-being also improved, mindfulness skills and life satisfaction increased during the intervention period compared to the control period. In addition, mental health and self-compassion improved during the intervention period and not during the control period, but the between period difference was not statistically significant.

Finally, self-reported ethical leadership behaviour increased during the course, compared to the control period, which was in particular due to improvement in role clarification (explaining what is expected of employees with regard to responsibilities and performance, and priorities) and ethical guidance (clarifying expectations regarding integrity, ensuring employees follow codes of integrity, stimulating discussion of integrity, complimenting employees on integer behavior).

Effect sizes were generally small to medium (0.3 to 0.6) and large for the increase in life satisfaction (0.8) (see Table 3).

### Qualitative study

Seventeen participants were interviewed, six males and eleven females, aged 40 to 57 years (see Table 4). The main themes that emerged from the interview data on the feasibility of completing the course are presented in Table 5 and discussed below.

**Table 4:** Characteristics of the participants of the qualitative study part

Identification number	Gender	Age	Specialty
1	Male	51–60	Supportive
2	Male	51–60	Internal
3	Female	51–60	Internal
4	Female	51–60	Supportive
5	Male	51–60	Surgical
6	Female	41–50	Supportive
7	Male	41–50	Internal
8	Female	41–50	Internal
9	Female	51–60	Supportive
10	Female	41–50	Surgical
11	Male	61–70	Surgical
12	Female	41–50	Surgical
13	Female	41–50	Internal
14	Male	61–70	Internal
15	Female	41–50	Internal
16	Female	41–50	Supportive
17	Female	31–40	Internal

**Table 5:** Barriers and facilitators defined as themes and subthemes as derived from codes that arose from the qualitative interviews

Main themes	Subthemes	Example quotes
1. Level of perceived benefit and appreciation of course contents	Facilitator subthemes Perceived benefit/ appreciation of	
	• Formal practice	*No.13: But then I was incredibly motivated! And I read everything, prepared everything, and did all of the exercises. And I thought it was very rewarding each time.*
	• Informal practice and practical application	*No.14: When I start or change an activity, the short two-, three-, four-minute body scan (...). Just taking a moment to sit still and listen to your body, your surroundings, or your breathing. That really helped me to get closure; to let go, or whatever you want to call it. And to prepare for what’s to come; to keep an open mind and think about what I’ll do next.*
	• Combination mindfulness with leadership, CBT, compassion	*No. 3: ...It’s kind of a two-part course: mindfulness on the one hand and leadership aspects on the other and ... that combination really appealed to me.... I wouldn’t have done just mindfulness, but the combination was really good...*
	• Group setting, sharing with colleagues, common humanity	*No. 6: I also liked being around like-minded people... That almost everyone... worked at Radboud, is roughly the same age, has a similar background and... struggles with the same day-to-day things and patient care. The daily grind, so to speak. So that created a... a kind of atmosphere of trust, which I really enjoyed and found useful*
	Barrier subthemes No perceived benefit/appreciation of	
	• Formal practice	*No. 6: No, so that sitting on a stool... that’s one way... but that’s going a step too far and something you really don’t feel like doing. So no, not me.* *No. 24: ... Slowly... no, I thought it was fine, but yeah, you just lie there on your back and tense and relax your muscles, and in a group setting... that didn’t really do much for me.*
	• Leadership component	*No. 4: So I was really enthusiastic about the first part of the course [mindfulness], and not so much about the second [leadership].*
2. Ability and willingness to spend time	Facilitator subthemes	
	• Motivation / need	*No. 5: Well because I thought: this is really important for me right now, and I wasn’t going to get there, no matter how hard I worked, because in the end that was exactly what I... with that never stopping.... it wouldn’t have gotten any better, despite how hard I was trying. So I thought: I’ve got to do this for myself and this should help. So I was extremely motivated.*
	• Flexibility working hours, no full time work (yet)	*No. 17: It was definitely feasible in that sense. I mean, it’s a serious time investment, of course, but I had the luxury of being in a kind of reintegration process at the time. And I was doing modified work, which made this easy to fit in.*
	• Integrating homework in daily life	*No. 5: ...I’ve become much more conscious in between... during my shifts... of bringing an apple or something with me, and taking a moment like halfway through to catch a break... because we don’t really have real break times, but to take a moment to sit down and eat...*
	• Practicalities	*No. 6: The impact, that is real, because it took so long, I think that it has a real impact...*
	Barrier subthemes	
	• No need or benefit	*No. 12: ...But I didn’t really feel the need and therefore didn’t really notice a difference. I’m a really happy person and ... I don’t have a lot of free time. I have small children at home and I have a job here that never really stops, that is, you know if that’s what you would want..... and if there is anything that is inconvenient, well investing so much time in.... inner peace... I just never felt like I had a problem for which this would be the solution.*
	• Limited time due to working more than full time	
	• Other priorities – children	
	• Practicalities – time/time on the day	*No. 3: Anyway, it’s tricky if you keep having to ask colleagues to take over your tasks.* *No. 5*: *I found it INCREDIBLY stressful... I think we started at 3 and I’d worked in the polyclinic from 8 to about 2... and with no chance to eat, and then I still had a lot to do before the weekend and so... before I could get there.... so I thought that was stressful.... but once I was there, I enjoyed it.*

#### Perceived benefits of course content

The extent to which interviewees appreciated and benefitted from the course content determined whether it was experienced as a facilitator or barrier. Formal practices, such as meditation, body scan and yoga were reported as both facilitators and barriers. Some reported to particularly appreciate the variety of practices and their experiential nature. Others found it hard to practice, especially to ‘slow down’ while experiencing high time pressure.

Most of the interviewees benefitted from the shorter informal mindfulness practices (such as breathing space, brief body scan, mindful eating) and the practical application of mindfulness in their daily work. For example, some reported to more consciously move from one activity to another by doing a brief body scan or a 3 min breathing space.

Many reported to appreciate the combination of mindfulness with elements of cognitive behavioral therapy, compassion and leadership. In particular integrating mindfulness into their daily leadership was considered helpful. For some the leadership component had been the reason to participate.

Most of the interviewees mentioned the group setting as a key facilitating factor. In particular being with other medical specialists and discussing experiences with each other, both during the sessions and the mealtimes, was seen as important. It helped them feeling less lonely in their experiences and connected with others.

#### Ability and willingness to spend time

Not surprisingly, the required time investment was often mentioned in the interviews, with the extent to which participants were able and willing to spend time determining whether the course was perceived as feasible. For some, their motivation and perceived need to change something strongly facilitated their participation. They deliberately choose to spend time on this, because they were returning to work from a burnout or other period of sick leave. In these situations, ‘not working full time yet’ was helpful. Others reported ‘flexibility in working hours’ as facilitating to make time for the course.

Quite some interviewees mentioned that they found practicing at home too time consuming. They mentioned to have practiced less than was recommended, in particular the formal mindfulness practices. Some did not consider it worthwhile to practice. A clear barrier was the limited time available because of long working hours or setting other priorities such as caring for children. Some found it hard to find a physical place to practice at home.

Other practical and organizational aspects were reported, such as the start of the course at 4 pm. Specialists sometimes had to find replacement or reported to have to work out of hours due to the missed time. The duration of the sessions themselves and the period of 20 weeks during which the course took place were reported to be facilitating factors to fundamentally change behaviour. It was also considered helpful that the course was offered in the hospital where most participants worked.

## Discussion

To our knowledge, this is the first study investigating the feasibility of a Mindful Leadership course for medical specialists and its effectiveness on burnout, well-being and leadership skills. We found that most aspects of burnout, well-being and leadership improved during the intervention period, even when compared to the control period. Furthermore, we found promising results regarding feasibility. Facilitators included perceived benefits, mindfulness practices and motivation. However, a major barrier was the limited amount of the time medical specialists have, due to high workload.

The results of our quantitative study indicate that a course in mindful leadership was associated with reduced burnout symptoms and improved psychological well-being. This confirms results from earlier studies on the effectiveness of mindfulness based interventions in mixed groups of health care personnel, which often included smaller groups of medical specialists, or did not include a control group or period [16]. Reduced burnout symptoms and increased psychological well-being is relevant for the medical specialists themselves, but is also known to be related to reduced sickness absence and improved job satisfaction of their staff [8], and in turn to increased patient satisfaction, patient safety and quality of care and possibly even treatment outcomes [7]. Furthermore, we found that a Mindful Leadership course resulted in improved self-reported competency in ethical leadership, which was not reported previously in a medical setting. These results are in line with previously suggested effects of mindfulness on leadership outside a medical setting, such as increased other-orientation (versus self-orientation) [12], higher quality relationships [12, 23] and achieving ethical goals [13].

When discussing feasibility, we must take into account that recruitment of medical specialists for this time intensive course was difficult. However, the low drop-out and high attendance rates indicate that for those who did decide to take part in this intensive course, participation was feasible. Appreciation of course elements, observing beneficial effects and motivation were major facilitators. However, difficulty of finding time in a high demand job with a high workload was a major barrier for participating, in particular for those with small children.

Most of the interviewees reported to have practiced less than was recommended, and mainly conducted the informal practices or applied mindfulness in their daily lives and work. Despite practicing less than recommended, most of the participants completed the course, and they overall reported positive effects. This raises the question if practicing at home for 30–45 min per day is a requirement to obtain effects of mindfulness courses, or if psycho-education in combination with shorter practices, informal practices and the recommendation to apply mindfulness in daily life would suffice. A recent systemic review reported a small but significant association between self-reported home practice and intervention outcomes, but also emphasized the necessity of further research on this topic [43].

Although the duration of the sessions and course were considered a barrier, the duration was also reported to facilitate learning mindfulness skills and applying these into their work. Alternative time schedules taking into account the perceived time pressure of medical specialists may increase the participation rate, but should take the time required for learning these mindfulness skills into account. It can be considered to provide the same contents in less sessions but over a comparable period of time, for instance by providing parts of the course online. Given the long working hours among medical specialists, the feasibility of participation might be improved if this is supported by the management of health care organizations, for instance by providing time during working hours to participate in courses or practice. This may require cultural changes in organizations, which further underscores the importance of training the management and medical leaders themselves.

Strengths of the study were the high completion and response rates, and the relatively large number of medical specialists included in the study. However, there were also limitations. Firstly, there was no randomization, and there was a low number of participants included in the control period. However, additional analyses including only participants who completed all three questionnaires showed results in the same direction and order of magnitude, although with less statistical power due to lower numbers. This indicates that it is unlikely that the results can be explained by differences between the participants of control and intervention period only. The low number of participants contributing to the control period is partly due to practical problems; participants often subscribed to the course shortly before the start of the course and too late to be included in the control period. However, it is also considered a strength of the study that at least control data were included, which is often not done in studies of medical specialists [17]. Secondly, because the intervention comprised multiple components, it cannot be determined to what extent the results of this study can be attributed to mindfulness itself or to the other components. Thirdly, the first two cohorts participated in the course for free, whereas the last two cohorts also received (partial) financial compensation through use of their personal development budget. This may have influenced our response rate and seriousness by which the respondents carried out this intervention. Other limitations included the self-report character of the effectiveness measures and the absence of follow-up data.

Although challenging in this setting, larger studies preferably using randomized designs with longer follow up periods would be needed to confirm our findings. Our findings particularly encourage further investigation of the effects of mindfulness on leadership skills in a medical setting, preferably using ‘observer reported’ data or data on quality of care. In addition, it would be of interest to explore in more detail how this course affected medical specialist’s leadership style, and in particular, what aspects of the course (i.e. mindfulness, cognitive behavioral therapy, compassion, leadership theories) are most helpful. Finally, our feasibility data point to the relevance of experimenting with different time schedules and to investigate the ‘minimal effective dose’ of mindfulness practice in future studies.

## Conclusions

In conclusion, a mindful leadership course for medical specialists was associated with reduced burnout symptoms, improved well-being and improved self-reported competency in ethical leadership. It was generally feasible to complete the course. Appreciation of course elements was a major facilitator and the difficulty of finding time a major barrier for participating. Although design limitations warrant careful interpretation, mindful leadership courses could be a valuable part of ongoing professional development programs for medical specialists.

## Data Availability

The datasets generated and analyzed during the current study are available from the corresponding author for inspection on reasonable request.

## Abbreviations

ELW: Ethical Leadership at Work questionnaire
LSQ: Life Satisfaction Questionnaire
MBSR: mindfulness-based stress reduction
MHC-SF: Mental Health Continuum - short form
PSWQ: Penn State Worry Questionnaire Survey
SCS-SF: Self-compassion scale- short form
SE: Standard error
SWING: Work Home Interaction Nijmegen scale
UBOS-C: Utrecht Burnout Scale
